# The epigenetic factor PCAF regulates vascular inflammation and is essential for intimal hyperplasia development

**DOI:** 10.1371/journal.pone.0185820

**Published:** 2017-10-10

**Authors:** Rob C. M. de Jong, Mark M. Ewing, Margreet R. de Vries, Jacco C. Karper, Antonius J. N. M. Bastiaansen, Hendrika A. B. Peters, Fabiana Baghana, Peter J. van den Elsen, Céline Gongora, J. Wouter Jukema, Paul H. A. Quax

**Affiliations:** 1 Department of Surgery, Leiden University Medical Center (LUMC), Leiden, The Netherlands; 2 Einthoven Laboratory for Experimental Vascular Medicine, Leiden University Medical Center (LUMC), Leiden, The Netherlands; 3 Department of Cardiology, Leiden University Medical Center (LUMC), Leiden, The Netherlands; 4 Department of Immunohematology and Blood Transfusion, Leiden University Medical Center (LUMC), Leiden, The Netherlands; 5 Department of Pathology, VU University Medical Center, Amsterdam, the Netherlands; 6 Université de Montpellier 2, Montpellier, France; 7 Institut de Recherche en Cancérologie de Montpellier INSERM U 89, Montpellier, France; Universita degli Studi di Padova, ITALY

## Abstract

**Objective:**

Genetic P300/CBP-associated factor (PCAF) variation affects restenosis-risk in patients. PCAF has lysine acetyltransferase activity and promotes nuclear factor kappa-beta (NFκB)-mediated inflammation, which drives post-interventional intimal hyperplasia development. We studied the contributing role of PCAF in post-interventional intimal hyperplasia.

**Methods and results:**

PCAF contribution to inflammation and intimal hyperplasia was assessed in leukocytes, macrophages and vascular smooth muscle cells (vSMCs) *in vitro* and in a mouse model for intimal hyperplasia, in which a cuff is placed around the femoral artery. PCAF deficiency downregulate CCL2, IL-6 and TNF-alpha expression, as demonstrated on cultured vSMCs, leukocytes and macrophages. PCAF KO mice showed a 71.8% reduction of vSMC-rich intimal hyperplasia, a 73.4% reduction of intima/media ratio and a 63.7% reduction of luminal stenosis after femoral artery cuff placement compared to wild type (WT) mice. The association of PCAF and vascular inflammation was further investigated using the potent natural PCAF inhibitor garcinol. Garcinol treatment reduced CCL2 and TNF-alpha expression, as demonstrated on cultured vSMCs and leukocytes.

To assess the effect of garcinol treatment on vascular inflammation we used hypercholesterolemic ApoE*3-Leiden mice. After cuff placement, garcinol treatment resulted in reduced arterial leukocyte and macrophage adherence and infiltration after three days compared to untreated animals.

**Conclusions:**

These results identify a vital role for the lysine acetyltransferase PCAF in the regulation of local inflammation after arterial injury and likely the subsequent vSMC proliferation, responsible for intimal hyperplasia.

## Introduction

Percutaneous coronary intervention (PCI) remains the main choice of revascularization therapy for coronary artery disease. However, intimal hyperplasia is a common complication and inflammation plays a pivotal role in its development [1–4]. Despite the introduction of (drug-eluting) stents, this problem remains in part of the patients. Endothelial injury during PCI promotes leukocyte attachment and extravasation [1, 3, 5]. Subsequently, leukocytes and vascular smooth muscle cells (vSMCs) produce pro-inflammatory cytokines which lead to vSMC migration, proliferation and extracellular matrix formation [1].

Nuclear factor kappa-beta (NFκB) is an important transcription factor which regulates the expression of many inflammatory related genes involved in cardiovascular disease [6]. Gene-environmental interactions that stimulate NFκB expression are regulated by epigenetic factors that strongly modulate gene expression patterns without DNA sequence modification, for example by regulating histone acetylation and de-acetylation [7, 8]. Inflammatory gene expression is the result of the counterbalancing and reversible actions of lysine acetyltransferases (KATs) and lysine deacetylases (KDACs), which together determine chromatin structure modification and accessibility to transcription factors [9].

P300/CBP associated factor (PCAF/KAT2B) is a transcriptional co-activator with intrinsic HAT-activity and is involved in lysine acetylation of histones at the site of NFκB-regulated genes [9–11]. Thereby PCAF regulates the NFκB-mediated increase in tumor necrosis factor (TNF)-alpha expression [10] and TNF-alpha regulates the inflammatory response that lead to intimal hyperplasia [12]. Previously, our group found that following hind limb ischemia PCAF-deficient mice differentially express 3505 genes in their adductor muscle group when compared to wild type mice [13]. Furthermore, Huang *et*. *al*. found that PCAF regulates the expression of inflammatory genes upon renal injury [14].

Previously, association between the -2481C variant allele of the PCAF gene and reduced vascular mortality was shown in three independent large prospective studies [15–17], identifying PCAF as possible diagnostic marker for CHD mortality and restenosis [18]. Increased intravascular *Pcaf* mRNA levels after injury suggested PCAF involvement in inflammatory-mediated remodelling, although the nature of this elevation remained unexplored [18]. Recently, it has been shown that PCAF expression was increased in abdominal aortic aneurysm tissue when compared to healthy aorta tissue [19].

Few natural inhibitors of PCAF have been described, of which only the natural inhibitor garcinol, derived from the Garcinia Indica fruit rind, has been shown to be extremely potent [20]. It inactivates PCAF activity rapid [21] and has strong apoptosis-inducing effect on leukemia cell lines [22], and also on prostate and pancreatic cancer cells [23] through inhibition of NFκB-DNA binding. These properties make garcinol an extremely potent inhibitor of PCAF-regulated inflammation, although garcinol may be not completely PCAF specific [24].

In the present study, the well characterized PCAF knock-out mice [25, 26] were used to investigate the contribution of PCAF to the inflammatory response following vascular injury in a reactive intimal hyperplasia mouse model [27, 28]. Furthermore, garcinol was used to investigate the effect of pharmaceutical PCAF inhibition on vascular inflammation in a hypercholesterolemic setting.

## Materials and methods

### Mice

This study was performed in compliance with Dutch government guidelines and the Directive 2010/63/EU of the European Parliament. All animal experiments were approved by the Institutional Committee for Animal Welfare of the Leiden University Medical Center (approval reference numbers 09094 and 09224). The generation of PCAF knockout (PCAF KO) mice has been described previously [29] and were kindly provided by Dr. C. Gongora. Male C57BL/6 PCAF KO mice and wild type (WT) C57BL/6 controls were used, as were transgenic male ApoE*3-Leiden mice (both bred in our own laboratory), backcrossed for more than 20 generations on a C57BL/6 background. ApoE*3-Leiden (at the start of a dietary run-in period) and WT and PCAF KO mice aged 10–12 weeks, were used for femoral artery cuff experiments.

### Diet

PCAF KO and WT mice received chow diet. Transgenic male ApoE*3-Leiden mice were fed a Western-type diet containing 1% cholesterol and 0.05% cholate to induce hypercholesterolemia (AB Diets). The diet was given three weeks prior to surgery and was continued throughout the experiment. All animals received food and water ad libitum during the entire experiment.

### Femoral artery cuff mouse model

To investigate the role of PCAF in intimal hyperplasia development, WT and PCAF KO mice underwent a non-constrictive cuff placement around the femoral artery to induce vascular inflammation and remodeling as previously described [27]. Mice were anesthetized before surgery with an i.p. injection of midazolam (8 mg/kg, Roche Diagnostics), medetomidine (0.5 mg/kg, Orion) and fentanyl (0.05 mg/kg, Janssen Pharmaceutica). To investigate short term inflammatory cell influx, cuff placement was performed on hypercholesterolemic ApoE*3-Leiden mice treated with garcinol or vehicle.

After 21 days (WT and PCAF KO mice) or after 3 days (ApoE*3-Leiden mice) mice were anesthetized as before and sacrificed via perfusion. The thorax was opened and pressure-perfusion (100mm Hg) with PBS was performed for 3 minutes by cardiac puncture of the left ventricle. After perfusion with 3.7% formaldehyde the cuffed femoral arteries were harvested, fixed for 5 hours in formaldehyde and paraffin-embedded.

### In vivo garcinol treatment

During non-constrictive cuff placement, ApoE*3-Leiden mice were treated with 10 μl pluronic gel F127 (40%, maintained at 0°C, Sigma Aldrich) ± 25 mg/ml garcinol (Enzo Life Sciences). In this way, garcinol was slowly released over a period of a couple of days at the site of injury. The pluronic gel with or without garcinol was lubricated around the isolated femoral artery and was allowed to harden out and settle around the cuff, which occurred within 20 seconds after application.

### Plasma analysis and ELISA

Total plasma cholesterol concentration (Roche Diagnostics, kit 1489437) was measured enzymatically. Inflammatory cytokine concentration of chemokine (C-C motif) ligand 2 (CCL2), interleukin-6 (IL-6) and TNF-alpha were determined using ELISA kits (555260, 555240 and 558534, Becton Dickinson), according to the manufacturer's instructions.

### (Immuno)histochemistry (IHC)

To detect the presence of inflammatory cells, vessel wall characteristics and effects of garcinol therapy, IHC was performed on paraffin-embedded sections of cuffs harvested after 3 days (ApoE*3-Leiden) or 21 days respectively (PCAF KO and WT mice). Weigert’s elastin staining was used to visualize elastic laminae. Inflammatory cell presence in the vascular wall was visualized using antibodies against leukocytes (anti-CD45 clone 30-F11, BD Pharmingen) and macrophages (anti-Mac3 clone M3/84, BD Pharmingen). Vascular SMCs were stained using anti-smooth muscle actin (SMA, clone 1A4, Dako) antibodies, PCAF was stained using anti-PCAF (ab12188, Abcam) antibodies and CCL2 was stained using anti-CCL2 (clone M-18, Santa Cruz) antibodies.

### Morphometric analysis

All quantifications in this study were performed on six equally spaced serial stained perpendicular cross-sections throughout the entire length of the vessel, as described previously [30]. Using image analysis software (Qwin, Leica), total cross-sectional medial area (between both elastic laminae), neointimal area (between internal elastic lamina and lumen) and luminal area was measured. These values were used to calculate the intima / media ratio and percentage luminal stenosis. In the short term study (3 days) the number of leukocytes, macrophages and CCL2 positive cells and after 21 days in PCAF KO vs. WT macrophages were counted manually and expressed as a percentage of the total number of cells (stained with hematoxylin). CCL2 and vSMC content after 21 days is analyzed using Qwin and CCL2 is expressed as the percentage of total medial and intimal area stained positive for CCL2. Vascular SMC content is expressed as both the percentage and the area (μm^2^) of total medial and intimal area stained positive for SMA.

### In vitro immune response

#### Whole blood-derived leukocytes

Blood was drawn from PCAF KO and WT mice via tail vein bleeding. The blood was diluted 1:25 with RPMI 1640 (Invitrogen) supplemented with 1% penicillin/streptomycin (Invitrogen). Blood was incubated in the presence and absence of lipopolysaccharide (LPS) from Escherichia coli K-235 L2018 (Sigma Aldrich) alone or together with garcinol. The cells were incubated overnight at 37°C in 5% CO_2_ atmosphere. After 24 hours incubation the supernatants were collected and analyzed by ELISA.

#### Vascular smooth muscle cells

Murine aortas were harvested from PCAF KO and WT mice. The aortas were cut longitudinally to expose the luminal side. The endothelial cells were removed by gently scraping. The aortas were cut in small pieces and placed on gelatin-coated culture dishes. The explants were cultured in DMEM (PAA laboratories) containing 20% FCS heat-inactivated (Lonza), 1% penicillin/streptomycin (Invitrogen) and 1% NEAA (PAA laboratories). Cells were cultured and used for experiments at passages 2 to 4. It should be noted that, by using this method, SMA positive myofibroblasts may be isolated as well. To evaluate the effects of inflammation on inflammatory cytokine expression, confluent layers of vSMC were seeded out in DMEM supplemented with 8% FCS heat-inactivated and 1% penicillin/streptomycin and cultured for 24 hours. Vascular SMCs were stimulated by exposure to 8% FCS heat-inactivated in the presence and absence of LPS alone or together with garcinol. The cells were incubated overnight at 37°C in 5% CO_2_ atmosphere. After 24 hours incubation the supernatants were collected and analyzed by ELISA.

#### *Pcaf* silencing in vSMCs

Vascular SMCs were transfected using Lipofectamine 2000 (Invitrogen) according to the manufacturer’s instructions with control short-interfering RNA (scrambled siRNA; Qiagen) or a combination of 4 siRNAs directed towards *Pcaf* (Qiagen) for 4 hours. Subsequently vSMCs were stimulated with LPS (1 ng/ml) for 24 hours and supernatants were collected and analyzed by ELISA. To confirm *Pcaf* knockdown, qPCR was used to analyze *Pcaf* expression. RNA was isolated using RNeasy minikits (Qiagen) and qPCR was performed on ABI7500 Fast system using Taqman gene expression assays for *Hprt1* and *Pcaf*.

#### Bone-marrow derived macrophages

Bone-marrow (BM) derived cells were isolated from PCAF KO and WT mice and subjected to murine macrophage colony-stimulating factor (M-CSF) (20 ng/μl; Miltenyi Biotec) to stimulate differentiation into macrophages.

Macrophages were stimulated by exposure to 8% FCS heat-inactivated in the presence and absence LPS alone or together with garcinol. The cells were incubated overnight at 37°C in 5% CO_2_ atmosphere. After 24 hours incubation the supernatants were collected and analyzed by ELISA.

### Cell viability assay

Since garcinol can affect cellular viability in high concentrations due to aspecific effects, garcinol-induced apoptosis in ex vivo whole blood was assessed in heparinized venous whole blood drawn from WT mice. Blood was diluted 1:25 in RPMI together with (0, 2.5, 10, 15, 20, 30, 50, 100 or 250 μM) garcinol for 24h at 37°C in 5% CO_2_ atmosphere. After 24h incubation, red blood cells were lysed and the medium was refreshed with RPMI supplemented with 8% FCS and 10% (vol/vol) Alamar blue (Invitrogen). The optical density of each well was measured in a Millipore CytoFluor 2300 plate-reading Fluor meter with excitation at 560 nm and emission at 615 nm when medium in untreated samples turned pink (±4h). Cell viability (%) was calculated compared with positive (untreated) control cells.

### Statistical analysis

Data are expressed as mean ± SEM. Two-tailed Student’s t-tests were used to compare groups. A level of *P*<0.05 was considered significant.

## Results

### PCAF deficiency leads to reduced inflammatory cytokine production *in vitro*

**Fig 1:**
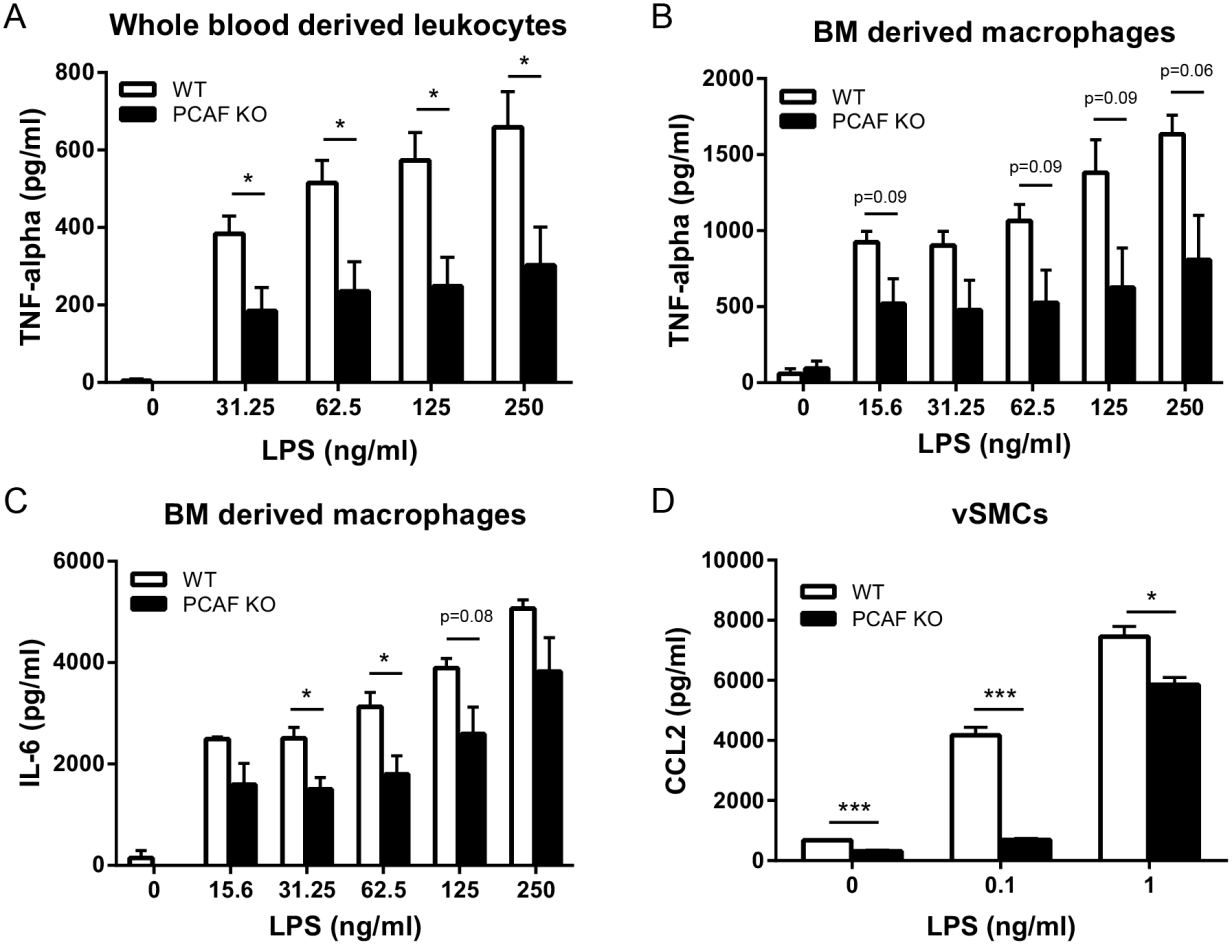
Effect of PCAF deficiency on inflammatory cytokine expression *in vitro*. (A) TNF-alpha production of whole blood derived leukocytes (n = 5) from WT and PCAF KO mice 24 hours after LPS (0–250 ng/ml) stimulation. *P<0.05. TNF-alpha(B) and IL-6 (C) production of bone marrow derived macrophages (n = 3) from WT and PCAF KO mice 24 hours after LPS (0–250 ng/ml) stimulation. *P<0.05. (D) CCL2 production of vascular smooth muscle cells (n = 3) from WT and PCAF KO mice 24 hours after LPS (0–1 ng/ml) stimulation. *P<0.05, ***P<0.001. Results are mean±SEM.

To investigate whether PCAF has a specific effect on cytokine production, we determined the effect of PCAF deficiency on inflammatory cytokine production *in vitro*. TNF-alpha production in whole blood derived leukocytes of PCAF KO mice was significantly reduced 24 hours after stimulation with LPS, when compared to whole blood derived leukocytes of WT mice (Fig 1A). Also in PCAF KO BM derived macrophages TNF-alpha production reduced compared to WT macrophages upon LPS stimulation although not significantly (Fig 1B), whereas IL-6 production was significantly reduced after stimulation with 31.25 and 62.5 ng/ml LPS (Fig 1C). CCL2 production of PCAF KO compared to WT vSMCs was already reduced without LPS stimulation (Fig 1D). This reduction was even bigger after stimulation with 0.1 and 1 ng/ml LPS.

### Intimal hyperplasia is reduced in PCAF KO mice

**Fig 2:**
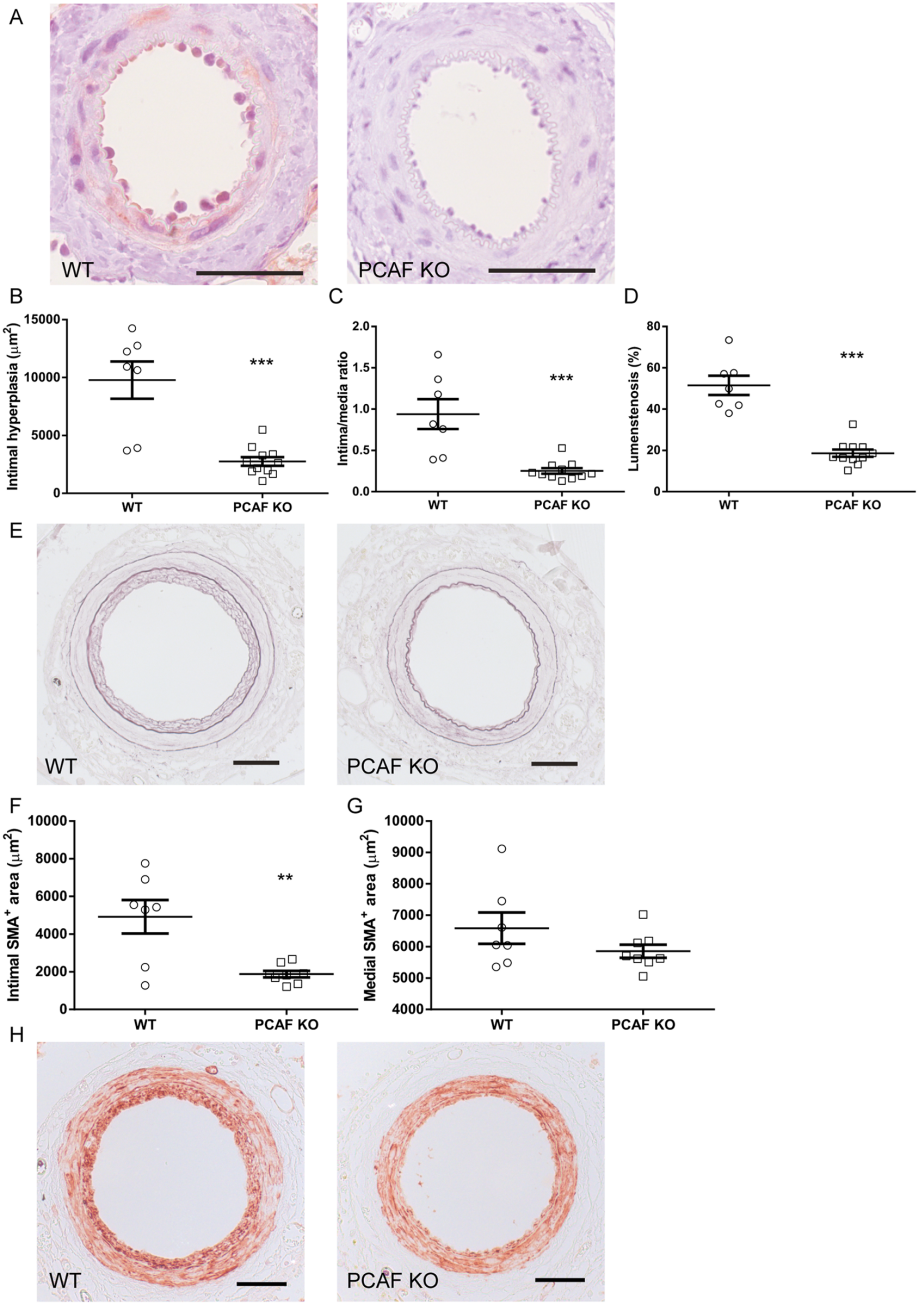
Effect of PCAF deficiency on intimal hyperplasia and vascular smooth muscle cell content *in vivo*. Representative images of PCAF staining (A), scale bar = 100 μm. Quantification of intimal hyperplasia (B), intima/media ratio (C) and lumenstenosis (D) 21 days after cuff placement in WT (n = 7) and PCAF KO (n = 11) mice. ***P<0.001. Representative images of elastin staining (E), scale bar = 50 μm. Quantification intimal (F) and medial (G) smooth muscle cell area (μm^2^) 21 days after cuff placement in WT (n = 7) and PCAF KO (n = 8) mice. **P<0.001. (H) Representative images of smooth muscle actin (SMA) staining of cuffed femoral arteries, scale bar = 50 μm. Results are mean±SEM.

To confirm PCAF deficiency of our PCAF KO mice we performed an immunohistochemical staining using specific anti-PCAF antibodies. As can be observed in Fig 2A, clear PCAF staining is present in the femoral artery segments of WT mice, while it is completely absent in the PCAF KO mice. Having shown a reduction of inflammatory cytokine production in PCAF deficient cells *in vitro*, we next demonstrated the effect of PCAF deficiency on intimal hyperplasia of cuffed femoral artery segments of C57Bl/6 control and PCAF KO mice using Weigert’s elastin staining. Quantitative analysis revealed a significant reduction of intimal hyperplasia of 71.8% in PCAF KO mice when compared to WT mice (Fig 2B and 2E). Since the media area was comparable between groups, this was accompanied by a significantly reduced intima/media ratio by 73.4% (Fig 2C) and luminal stenosis by 63.7% after 21 days (Fig 2D).

The lesion of both the WT as the PCAF KO mice mainly consists of vSMCs as can be observed in Fig 2H. Total area of intimal SMA expressing cells was significantly decreased in PCAF KO mice compared to WT mice (Fig 2F), whereas total area of the medial SMA expressing cells was not affected (Fig 2G). To exclude a difference in vSMC content at baseline, uncuffed arteries of both the WT and PCAF KO mice were stained for SMA, and no difference in vSMC content was observed (S1A Fig). The reduced vSMC accumulation in the intima suggests a reduced vSMC migration and/or proliferation in PCAF KO mice. However, the number of Ki67 positive vSMCs was not reduced in both de media (S1B Fig) and intima (S1C Fig) in PCAF KO mice when compared to WT mice. We believe this is due to the time point of 21 days, which might be too late to analyze vSMC proliferation.

### PCAF deficiency does not lead to reduced macrophage influx and CCL2 expression after 21 days

**Fig 3:**
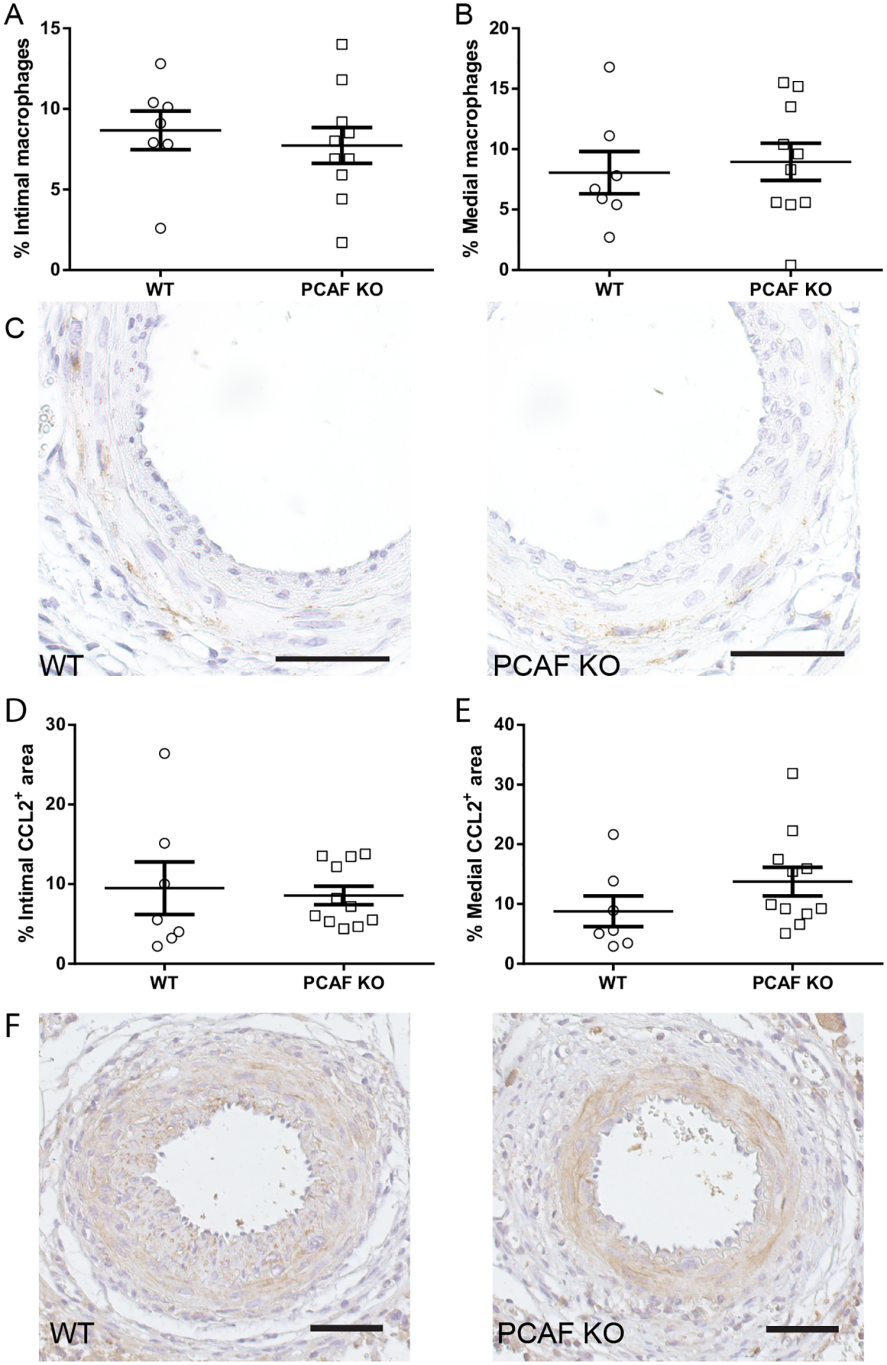
Effect of PCAF deficiency on macrophage influx and CCL2 expression *in vivo*. Quantification of Mac3 positive cells (macrophages) in the intima (A) and media (B) 21 days after cuff placement in WT (n = 7) and PCAF KO (n = 11) mice. Representative images of Mac3 staining (C), scale bar = 50 μm. Quantification of CCL2 positive area in the intima (D) and media (E) 21 days after cuff placement in WT (n = 7) and PCAF KO (n = 11) mice. Representative images of CCL2 staining (F), scale bar = 50 μm. Results are mean±SEM.

Next, we investigated the effect of PCAF deficiency on macrophage influx and CCL2 expression. The percentage of intimal and medial macrophages was not affected in PCAF KO mice compared to WT mice (Fig 3A, 3B and 3C). The percentage of intimal and medial CCL2 expressing cells was also not affected by PCAF deficiency (Fig 3D, 3E and 3F). Furthermore, medial and intimal TNF-alpha expression was not affected in PCAF KO mice compared to WT mice (S1D Fig).

The inflammatory response following vascular injury is a process that mainly takes place the first couple of days after injury [31]. Analysis of the inflammatory response at 21 days after cuff placement may be too late. Therefore, we investigated the short term inflammatory response using the pharmalogical PCAF inhibitor garcinol, in hypercholesterolemic ApoE*3-Leiden mice, in which the inflammatory response is more explicit.

### Garcinol inhibits inflammatory cytokine production *in vitro*

**Fig 4:**
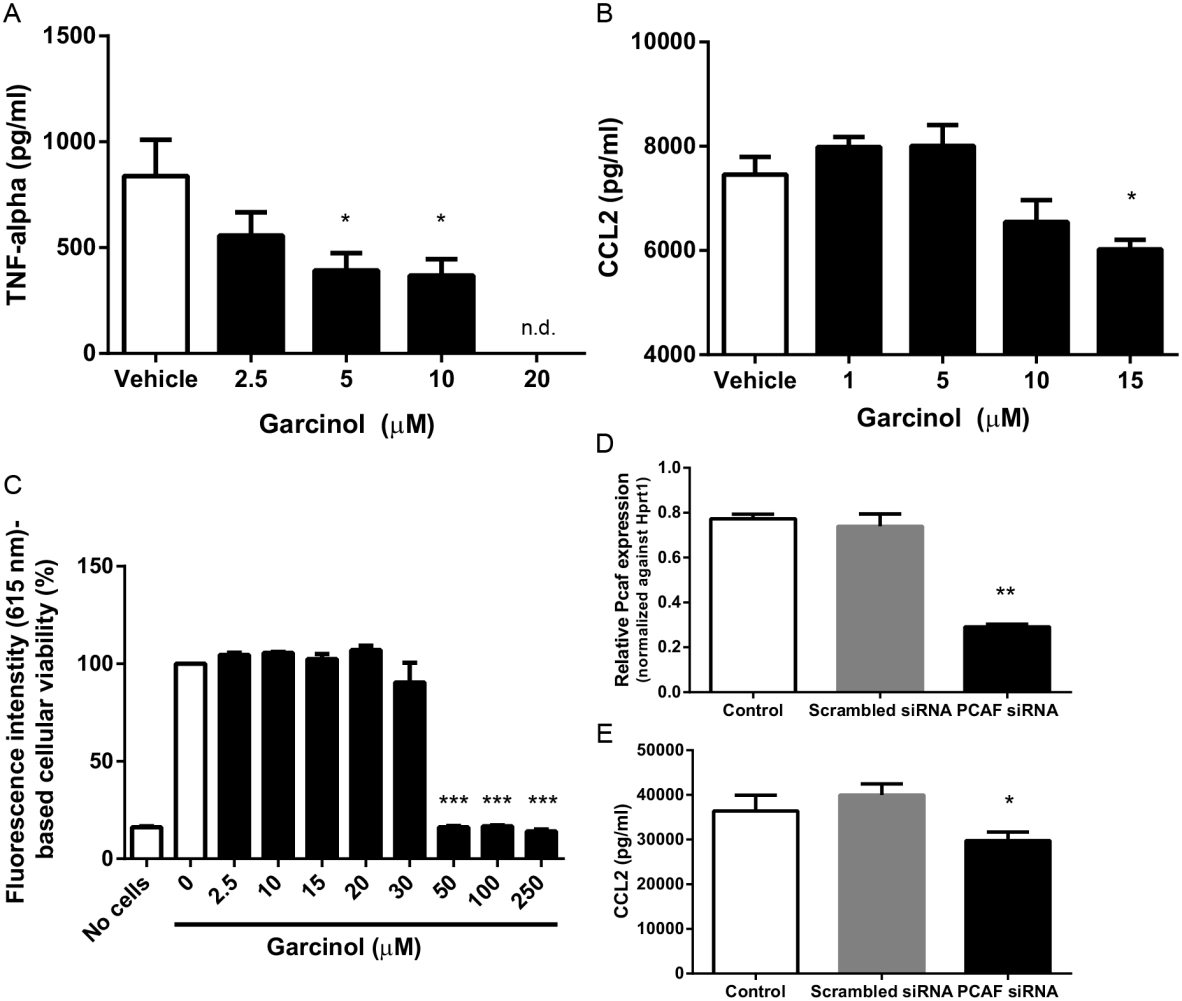
Effect of garcinol treatment and PCAF downregulation on inflammatory cytokine expression *in vitro*. (A) TNF-alpha production of whole blood derived leukocytes (n = 5) after 24 hours stimulation with LPS (100 ng/ml) in combination with garcinol (0–20 μM). *P<0.05 compared to vehicle (0 μM garcinol), n.d.: non-detectable. (B) CCL2 production of vascular smooth muscle cells (n = 3) after 24 hours stimulation with LPS (1 ng/ml) in combination with garcinol (0–15 μM). *P<0.05 compared to vehicle (0 μM garcinol). (C) Whole blood derived leukocyte viability (n = 4) after 24 hours incubation with garcinol (0–250 μM), expressed as percentage fluorescence intensity. ***P<0.001 compared to 0 μM garcinol. (D) Relative Pcaf expression of vascular smooth muscle cells (n = 3) transfected with scrambled or PCAF siRNA and stimulated with 1 ng/ml for 24 hours. **P<0.01 compared to scrambled siRNA. (E) CCL2 production of vascular smooth muscle cells (n = 3) transfected with scrambled or PCAF siRNA and stimulated with 1 ng/ml for 24 hours. *P<0.05 compared to scrambled siRNA. Results are mean±SEM.

First, we studied the effect of garcinol (0–20 μM) on TNF-alpha production of whole blood derived leukocytes stimulated with LPS (100 ng/ml). TNF-alpha production was significantly reduced when treated with 5 and 10 μM garcinol compared to vehicle (Fig 4A) and TNF-alpha production was totally abolished when treated with 20 μM garcinol. CCL2 production of vSMCs was reduced by 19.2% after LPS stimulation (1 ng/ml) in combination with garcinol treatment (15 μM) compared to vehicle (Fig 4B), but not when treated with lower concentrations of garcinol.

To study possible toxic effects of garcinol, we studied the viability of circulating leukocytes (whole blood) in the presence of garcinol (0–250 μM) using the redox indicator Alamar blue. Fluorescence intensity (FI) remained constant at ~5700 AU (615 nm) at garcinol concentrations 0–20 μM, indicating complete cellular viability comparable to positive controls (Fig 4C), with cytotoxicity at concentrations ≥30 μM (FI: ~900 AU). Furthermore, we used a MTT assay to study the possible toxic effects of garcinol on vSMCs, no detrimental effects of garcinol were found in concentrations range up to 20 μM (S2 Fig).

To exclude non-specific effects of garcinol treatment on vSMCs, we transfected vSMCs with specific *Pcaf* siRNAs to induce Pcaf knockdown. After transfection qPCR analysis revealed a significant knockdown of *Pcaf* (Fig 4D). CCL2 production was reduced by 25.6% following siRNA-mediated *Pcaf* knockdown compared to vSMCs transfected with a non-specific scrambled siRNA (Fig 4E), suggesting similar effects as observed after garcinol treatment. Moreover, we stimulated PCAF KO VSMCs simultaneously with LPS and 15 μM garcinol or vehicle and measured CCL2 production and no difference in CCL2 production is observed between both groups (S3 Fig). This again suggests that garcinol is, at least in the concentration range we use, specific for garcinol.

### Pharmacological PCAF inhibition reduces injury-induced leukocyte recruitment *in vivo*

**Fig 5:**
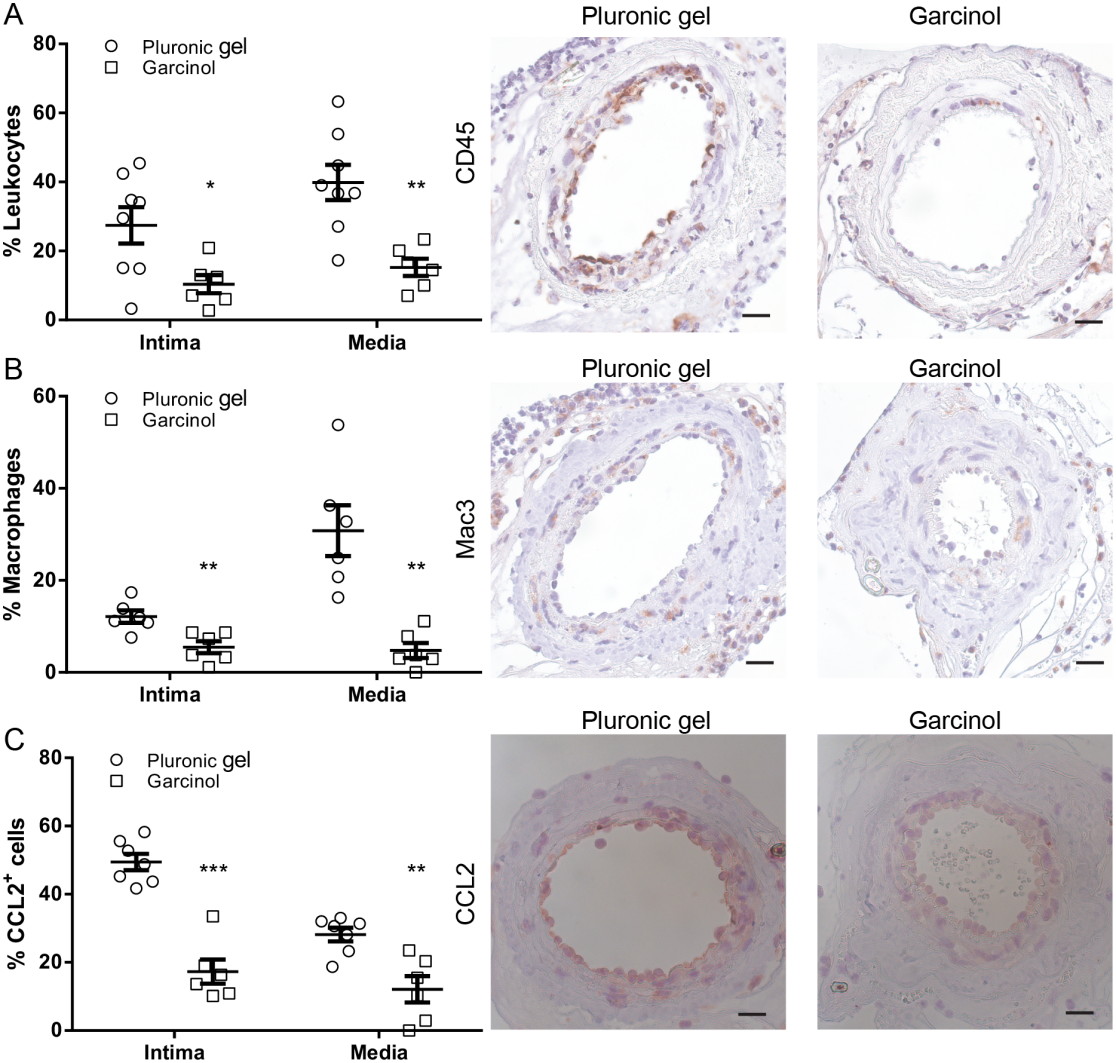
Effect of garcinol treatment on inflammatory cell recruitment and CCL2 expression *in vivo*. (A) Quantification of CD45 positive cells (leukocytes) in the intima and media 3 days after cuff placement in ApoE*3-Leiden mice treated with garcinol (n = 6) or pluronic gel (n = 8). *P<0.05, **P<0.01. (B) Quantification of Mac3 positive cells (macrophages) in the intima and media 3 days after cuff placement in ApoE*3-Leiden mice treated with garcinol (n = 6) or pluronic gel (n = 6). **P<0.01. (C) Quantification of CCL2 positive cells in the intima and media 3 days after cuff placement in ApoE*3-Leiden mice treated with garcinol (n = 6) or pluronic gel (n = 7). **P<0.01, ***P<0.001. Representative images of CD45, Mac3 and CCL2 staining of cuffed femoral arteries, scale bar = 20 μm. Results are mean±SEM.

Since garcinol reduces cytokine production *in vitro*, we treated hypercholesterolemic ApoE*3-Leiden mice with garcinol and investigated leukocyte infiltration 3 days after cuff placement. Short term garcinol treatment significantly reduced intimal leukocytes by 62.2% and medial leukocytes by 60.7% (Fig 5A) after 3 days. Since most of the leukocytes associated with the vessel wall are of the macrophage subtype, we also quantified these specifically. Intimal macrophages were reduced by 54.8% whereas medial macrophages were reduced by 84.5% compared to the pluronic gel control group (Fig 5B). Next, we studied the effect of garcinol on CCL2 expression, an important chemokine involved in attracting macrophages to sites of injury. Short term garcinol treatment reduced the percentage of cells in the intima that expressed CCL2 by 65.0% and of cells in the media by 57.0% (Fig 5C), suggesting that garcinol reduced leukocyte infiltration by affecting chemo-attracting factors.

## Discussion

In this study we demonstrate that PCAF deficiency reduces the *in vitro* inflammatory response and reduces development of vSMC-rich intimal hyperplasia *in vivo*. The natural PCAF inhibitor garcinol reduces the inflammatory response *in vitro*. Furthermore, we demonstrate that garcinol treatment reduces short term (3 days) leukocyte infiltration and CCL2 expression *in vivo*, following vascular injury in hypercholesterolemic apoE*3-Leiden mice. To our knowledge, this is the first paper to show that PCAF has a contributing role in intimal hyperplasia development, by regulating the inflammatory response and perhaps reducing the subsequent vSMC proliferation.

We used PCAF KO mice to demonstrate clear effects of PCAF deficiency on inflammatory-regulated intimal hyperplasia. Animals developed significantly smaller vSMC-rich lesions with reduced lumenstenosis. PCAF KO cells were used to demonstrate its essential contribution in the production of pro-inflammatory cytokines (CCL2, IL-6 and TNF-alpha) by various cell types including whole blood-derived leukocytes, BM derived macrophages and vSMCs. These results demonstrate an important role for the epigenetic factor PCAF in the post-interventional arterial inflammatory response.

TNF-alpha production was severely compromised in whole blood-derived leukocytes from PCAF deficient animals and in WT whole blood derived leukocytes treated with garcinol. In BM derived macrophages TNF-alpha production was also decreased, although not significantly. Previously it has been shown that lack of TNF-alpha in mice reduces intimal hyperplasia development [12, 32]. TNF-alpha production is regulated by NFκB [6] and PCAF is an important co-activator of NFκB [10]. Therefore, it is likely that the reduced intimal hyperplasia development in PCAF KO mice is, at least in part, caused by reduced TNF-alpha production.

IL-6 production in BM-derived macrophages was significantly decreased in PCAF KO macrophages compared to WT macrophages. Macrophages play an important role in vascular inflammation and one of the cytokines macrophages produce during vascular inflammation is IL-6 [33]. Furthermore, it has been shown that IL-6 recruits bone marrow cells to the vessel wall and thereby contributes to intimal hyperplasia [34]. Niida *et al*. showed that nuclear factor of kappa light polypeptide gene enhancer in B-cells inhibitor, delta (IκBNS) inhibits NFκB activity and IL-6 production in a mouse model for restenosis, subsequently leading to reduced intimal hyperplasia development [35]. Since PCAF is a coactivator of NFκB, and thus regulates IL-6 production, it is plausible that the reduced intimal hyperplasia is in part due to reduced IL-6 production.

PCAF deficiency led to reduced CCL2 production in vSMCs *in vitro*. These results were similar in wildtype cells treated with garcinol or subjected to siRNA mediated PCAF knockdown. Furthermore, garcinol treatment reduced CCL2 expression *in vivo* following vascular injury. Previous studies have shown that CCL2 plays an important role in the development of intimal hyperplasia, by regulation of the inflammatory response and vSMC proliferation [36–38]. In agreement, we found a reduced vSMC-rich intimal lesion size in PCAF KO mice compared to WT mice, which may indicate reduced vSMC proliferation. It is plausible that the effect of PCAF deficiency on CCL2 production is in part responsible for the reduced intimal hyperplasia development. Whether PCAF deficiency acts directly on vSMC proliferation or indirectly via inflammation or a combination of both, remains to be investigated.

Although we found a reduction in inflammatory cytokine production *in vitro* and reduced intimal hyperplasia development upon PCAF deficiency, we could not show a reduction in macrophage influx and CCL2 expression *in vivo*. Most likely this is due to the non-optimal time point to evaluate macrophage influx and CCL2 expression in PCAF KO mice. To overcome this problem we repeated the experiment and evaluated vascular inflammation at an earlier time point in a mouse model in which vascular inflammation is more profound, namely hypercholesterolemic ApoE*3-Leiden mice. Moreover, most patients suffering from coronary artery disease experience elevated cholesterol levels or hypercholesterolemia, which is an well-known risk factor for cardiovascular disease in human [39]. Therefore, we studied the effect of garcinol treatment in hypercholesterolemic ApoE*3-Leiden mice, mimicking the clinical situation regarding cholesterols levels. We found reduced leukocyte infiltration and CCL2 expression 3 days after cuff placement in hypercholesterolemic mice treated with garcinol. These results suggest that PCAF inhibition by garcinol reduces vascular inflammation in a clinical relevant mouse model. One should keep in mind that garcinol could affect other *in vivo* targets next to PCAF, like cyclooxygenase-2 (COX-2) [40], 5-lipoxygenase (5-Lox) [41] and others [24], that might contribute to the observed anti-inflammatory effect of garcinol in our study.

PCAF is involved in regulation of NFκB-mediated transcription of inflammatory genes in two different ways. First, PCAF is capable of acetylation of histone proteins at the site of NFκB-regulated genes, making the DNA at that specific site more accessible for NFκB. Second, PCAF is able to acetylate lysine residues on the p65 unit of NFκB itself, increasing its binding to the DNA [9]. Both mechanisms lead to an increase in transcription of pro-inflammatory genes, like CCL2, TNF-alpha and IL-6. We believe that both these mechanisms are affected in the PCAF KO mice and following garcinol treatment, leading to a decreased expression of pro-inflammatory genes and subsequently attenuation of intimal hyperplasia.

In conclusion, using PCAF KO mice, evidence is provided that PCAF contributes to post-interventional intimal hyperplasia. This could be explained by an as yet uncharacterized direct or indirect effect of PCAF on inflammation and vSMC proliferation, leading to reduced post-interventional intimal hyperplasia. These results shed light on the possible contribution of PCAF as an important epigenetic factor in intimal hyperplasia development in human coronary lesions and identify it as a possible new clinical target against intimal hyperplasia after PCI.

## Supporting information

S1 Fig Baseline vSMC content and effect of PCAF deficiency on vSMC proliferation and TNF-alpha expression. Representative images of SMA staining in uncuffed arteries (A). Scale bar = 20 μm. Quantification of Ki67/SMA^+^ cells in the media (B) and neointima (C) of WT (n = 7) and PCAF KO (n = 9) mice. Results are mean±SEM. Representative images of TNF-alpha staining of cuffed femoral arteries (D). Scale bar = 50 μm.
(TIF)

S2 Fig Effect of garcinol on VSMC apoptosis *in vitro*. MTT assay assessed viability of VSMCs stimulated with different concentrations garcinol. Results are mean±SEM.
(TIF)

S3 Fig Effect of garcinol on CCL production of PCAF deficient vSMCs. CCL2 production of vascular smooth muscle cells (n = 3) from WT and PCAF KO mice 24 hours after simultaneous LPS (0–0.1 ng/ml) and garcinol (0–15 μM) stimulation. Results are mean±SEM.
(TIF)

S4 Fig No primary antibody controls. Representative images of different no primary antibody controls. Scale bar = 50 μm.
(TIF)

S1 Text Supporting information. (DOCX)
