# Supplementary figures and images for: The epigenetic factor PCAF regulates vascular inflammation and is essential for intimal hyperplasia development
Source: PLoS One. 2017 Oct 10;12(10):e0185820. doi: 10.1371/journal.pone.0185820 (PMC5634597; doi:10.1371/journal.pone.0185820)

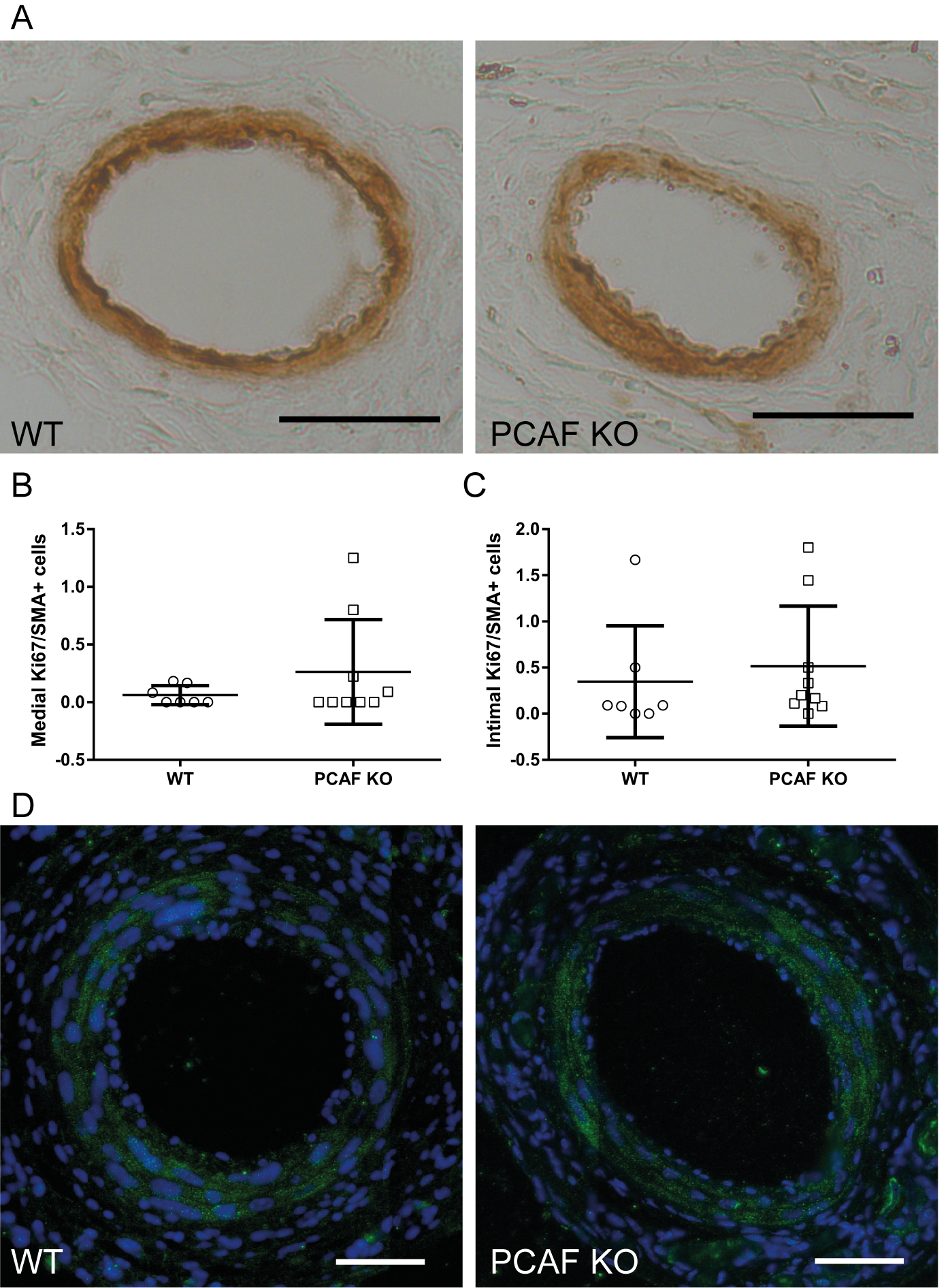

Supplement: S1 Fig — Representative images of SMA staining in uncuffed arteries (A). Scale bar = 20 μm. Quantification of Ki67/SMA+ cells in the media (B) and neointima (C) of WT (n = 7) and PCAF KO (n = 9) mice. Results are mean±SEM. Representative images of TNF-alpha staining of cuffed femoral arteries (D). Scale bar = 50 μm. (TIF) [file pone.0185820.s001.tif]

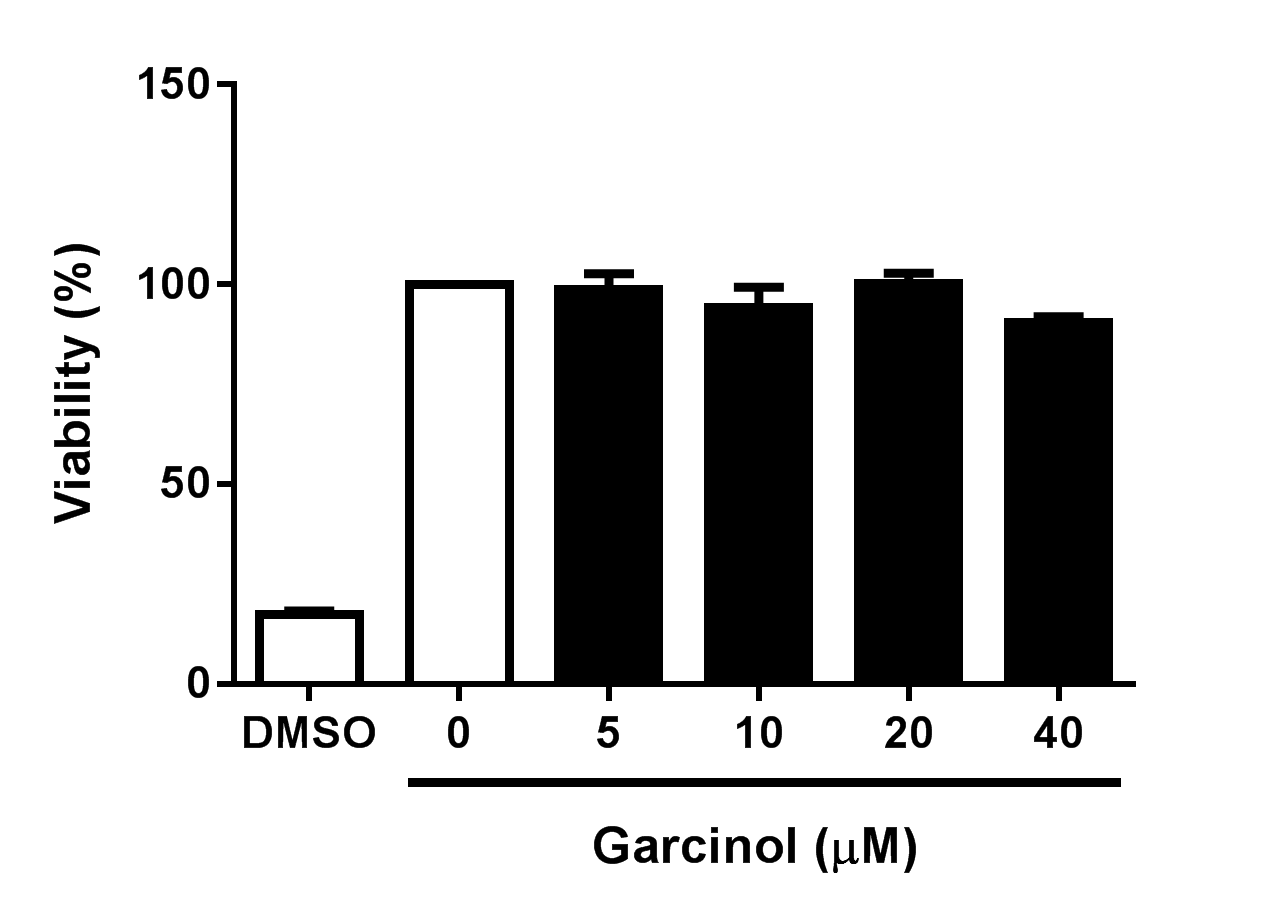

Supplement: S2 Fig — MTT assay assessed viability of VSMCs stimulated with different concentrations garcinol. Results are mean±SEM. (TIF) [file pone.0185820.s002.tif]

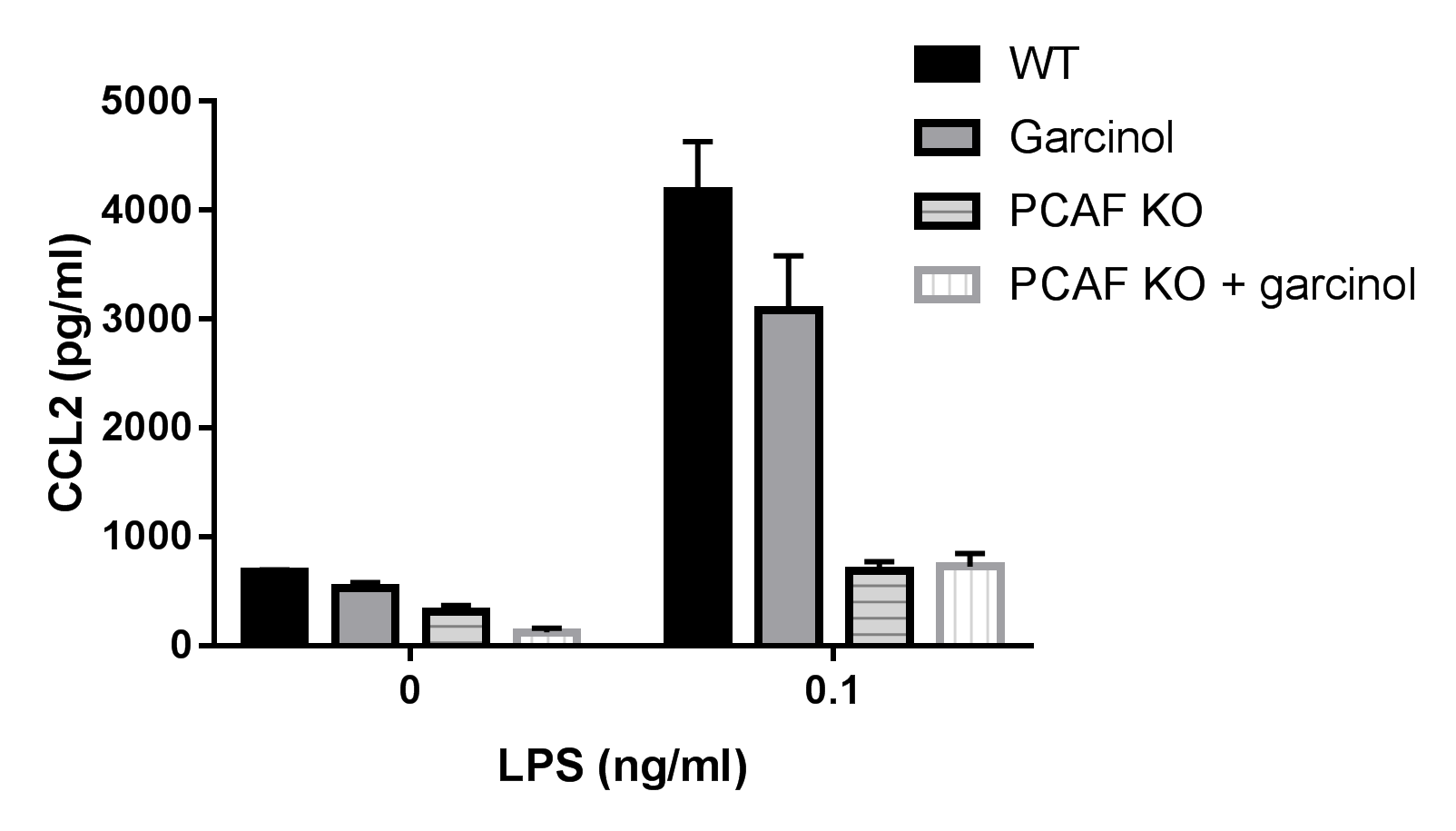

Supplement: S3 Fig — CCL2 production of vascular smooth muscle cells (n = 3) from WT and PCAF KO mice 24 hours after simultaneous LPS (0–0.1 ng/ml) and garcinol (0–15 μM) stimulation. Results are mean±SEM. (TIF) [file pone.0185820.s003.tif]

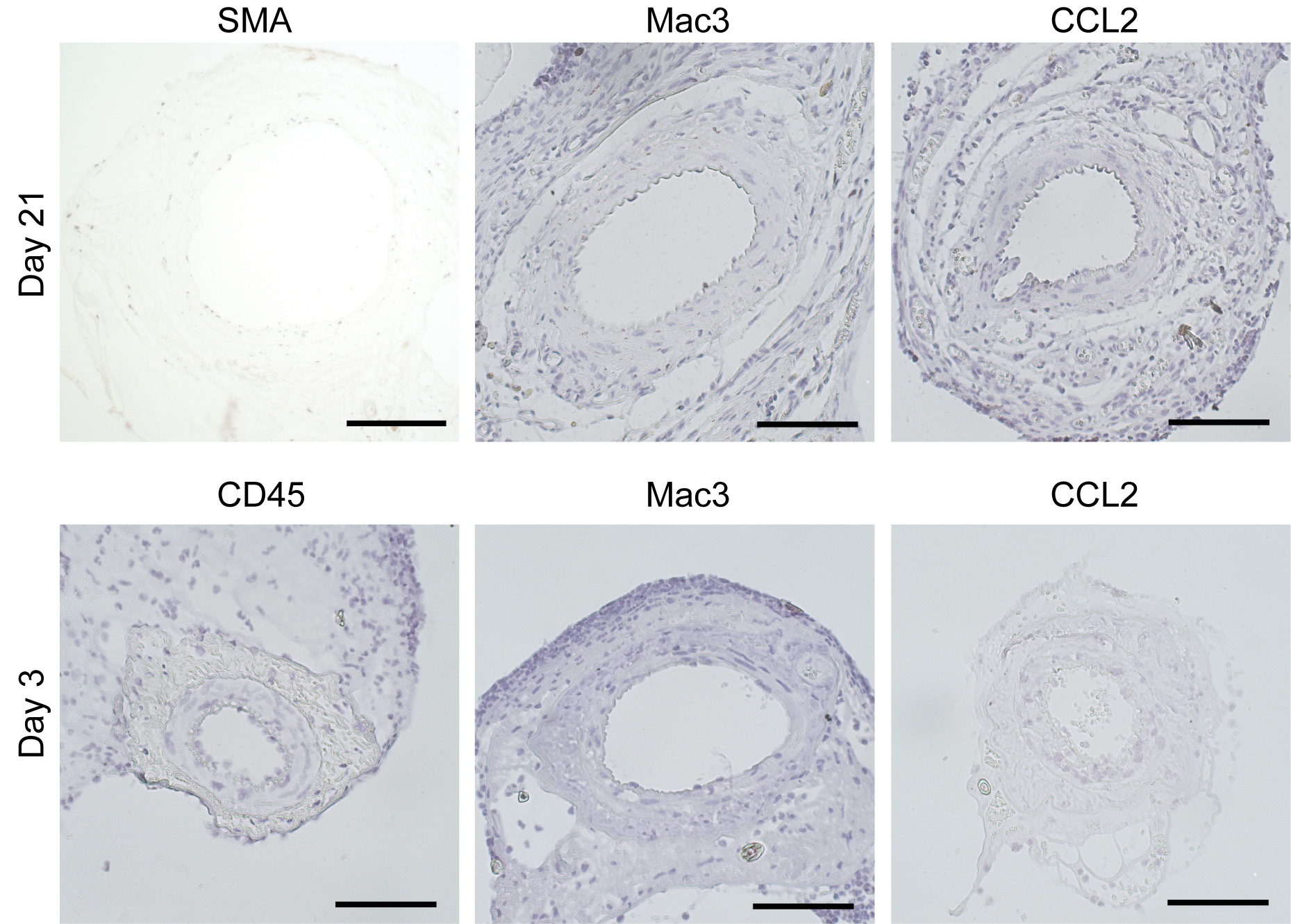

Supplement: S4 Fig — Representative images of different no primary antibody controls. Scale bar = 50 μm. (TIF) [file pone.0185820.s004.tif]
