# Supplementary material for: The epigenetic factor PCAF regulates vascular inflammation and is essential for intimal hyperplasia development
Source: PLoS One. 2017 Oct 10;12(10):e0185820. doi: 10.1371/journal.pone.0185820 (PMC5634597; doi:10.1371/journal.pone.0185820)
Supplement: S1 Text — (DOCX) [file pone.0185820.s005.docx]

**Supporting information**

**Supplemental materials & methods**

(Immuno)histochemistry (IHC)

IHC was performed on paraffin-embedded sections of uncuffed arteries or cuffed arteries of PCAF KO and WT mice harvested after 21 days. Vascular SMCs (vSMCs) were stained using anti-smooth muscle actin (SMA, clone 1A4, Dako) antibodies, while proliferating vSMCs were stained using the above mentioned anti-SMA antibodies in combination with anti-Ki67 (Abcam; ab16667) antibodies. TNF-alpha was stained using anti-TNF-alpha antibodies (Abcam; ab6671).

Quantification of proliferating vSMCs

Quantification of Ki67/SMA cells in media (left) and intima (right) of cuffed vessel segments, expressed as mean number per section , minimial six sections per cuffed segment.

MTT assay

At day zero 5.0^E^+03 VSMCs were seeded in a 96-wells plate in DMEM supplemented with 10% FCS heat-inactivated and 1% penicillin/streptomycin and cultured for 24 hours. The next day the medium was refreshed with DMEM supplemented with 10% FCS heat-inactivated and 1% penicillin/streptomycin with different concentrations (0, 5, 10, 20 , 40 µM) garcinol or 20% DMSO as negative control. After 24 hours incubation at 37°C, 10 µl MTT (12 mM) was added directly to the medium. After 4 hours incubation at 37°C, isopropanol was added and incubated for 1.5 hour on a shaker. Optical density was measured at 540 nm and cell viability (%) was calculated compared with positive (untreated) control cells.

In vitro effect of garcinol on PCAF deficient vSMCs

PCAF KO VSMCs were simultaneously stimulated with 0.1 ng LPS and 15 µM garcinol or vehicle for 24 hours and subsequently CCL2 levels were measured using ELISA kit (555260, Becton Dickinson), according to the manufacturer's instructions.
